# What does an explanted PASCAL device look like?

**DOI:** 10.1093/icvts/ivab318

**Published:** 2021-11-18

**Authors:** Mathias Van Hemelrijck, Juri Sromicki, Martin O Schmiady, Carlos-A Mestres

**Affiliations:** Clinic for Cardiac Surgery, University Hospital Zurich, Zurich, Switzerland

**Keywords:** Transcatheter therapy, Mitral regurgitation, PASCAL device

## Abstract

We report the case of a 78-year-old female patient who had a PASCAL device implanted for severe degenerative mitral regurgitation. Intraprocedural echocardiography revealed persistent severe mitral regurgitation due to device dislocation. Implanting another device was not possible. After 8 days, the device was explanted, and the valve was replaced with a biological prosthesis. The PASCAL device and resected mitral valve leaflets were sent for histopathological workup.

## INTRODUCTION

Transcatheter mitral valve repair is an alternative to surgery in symptomatic patients with secondary mitral valve regurgitation (MR) who are at high risk or deemed inoperable. Percutaneous treatment in degenerative MR is still debated [1]. We present the case of a patient initially treated with the PASCAL device (Edwards Lifesciences, Irvine, CA, USA) who required surgical treatment due to severe residual MR a few days after the transcatheter attempt.

## CASE REPORT

**Figure 1:**
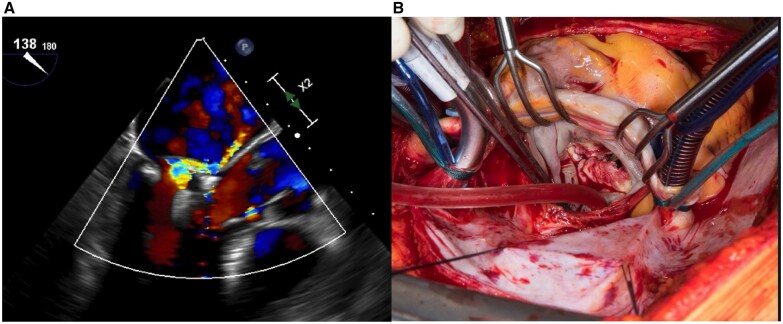
(**A**) Transoesophageal echocardiography: 3-chamber view showing severe mitral regurgitation after device implantation. (**B**) Surgeon’s view of the mitral valve. The arrow points to the torn posterior leaflet.

**Figure 2:**
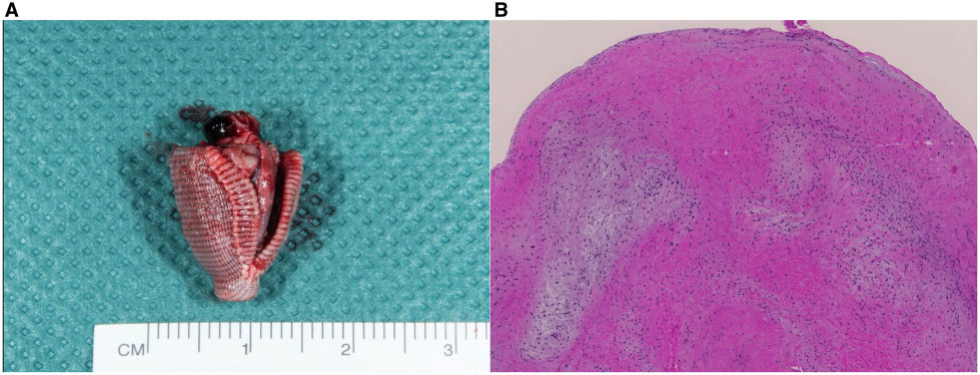
(**A**) PASCAL device after explantation. (**B**) Mitral valve tissue stained with haematoxylin and eosin. The image shows degenerative valve tissue and no signs of acute inflammation.

A 78-year-old female patient was diagnosed with severe symptomatic (New York Heart Association functional class III) degenerative MR with posterior prolapse and a flail leaflet in the P2 segment due to chordal rupture. Transthoracic echocardiography showed a calcified mitral annulus, a preserved left ventricular ejection fraction (62%) and no concomitant tricuspid regurgitation. The heart team recommended mitral valve surgery, but the patient refused the operation even though she had no comorbidities (EuroSCORE II 1.86%). She was offered transcatheter intervention with the PASCAL device. Implanting the device was technically uneventful; however, persistent severe MR was observed intraoperatively due to device-leaflet detachment (Fig. 1A). Surgical explantation was performed 8 days later through a median sternotomy. Cardiopulmonary bypass was established using bicaval-aortic cannulation. After cross-clamping, the heart was arrested using cold blood cardioplegia; the left atrium was approached through the Waterston-Sondergaard groove and the mitral valve was exposed. The PASCAL device was attached only to the anterior leaflet, and there was a tear in the posterior leaflet; the subvalvular apparatus was intact. The valve had extensive leaflet damage; hence it was unsuitable for reconstruction (Fig. 1B). The valve was excised, and the PASCAL device was explanted (Fig. 2A). A 29-mm bioprosthesis (St. Jude Medical Epic Valve, St Jude Medical, St. Paul, MN, USA) was implanted, leaving the subvalvular apparatus in place. Cross-clamp and cardiopulmonary bypass times were 83 and 115 min. On gross examination, the PASCAL device was intact. No superficial deposit of thrombus material was detected. A histopathological study showed degenerative valve tissue (Fig. 2B). The postoperative course was uneventful, and the patient was discharged to a rehabilitation clinic 10 days after the operation. Nine months after surgery, the patient is asymptomatic.

## DISCUSSION

Transcatheter mitral repair is an alternative to mitral valve surgery in selected cases [2, 3]. The MitraClip device (Abbott Vascular, Santa Clara, CA, USA) has been the only device on the market for a long time. The CLASP (Edwards PASCAL Transcatheter Mitral Valve Repair System) study [4] has shown acceptable 1-year outcomes after implanting the PASCAL device. The fourth generation (G4) MitraClip and the PASCAL device differ in design and in characteristics. The latter has a spacer between the arms that fills the regurgitation orifice, contributing to a broader leaflet insertion. The wide paddles minimize leaflet tension and navigation allows direct manoeuvring in 3 planes. Surgical experience with explanting the PASCAL device is scanty. Gerçek *et al.* [5] reported the first surgical explantation of this device in 2020 due to interventional failure. They concluded that surgical valve repair is still feasible after removing the device shortly after the initial implant. In our case, the operation was performed after 8 days, which is very early compared to unpublished data from the CUTTING-EDGE registry, which reported a median time to surgery of 4.8 months. As the time following device implantation increases, valve repair may become more demanding due to progressive tissue reaction and ingrowth. However, reparability not only depends on the initial valve pathology but also on lesions created by the device. As suggested by Gerçek *et al.*, device removal in the presented case was properly performed without causing any further damage to the valve apparatus. However, multiple grasping attempts at the index procedure may have led to valve tissue attrition.

## CONCLUSION

Even if the native valve structure is preserved, mitral valve repair after implanting a PASCAL device can be challenging. Valve reparability depends on the initial valve pathology, additional tissue damage incurred when the device was implanted, device ingrowth, surgeon experience and time after the device is implanted. Because the latter has a significant impact, the operation should not be delayed.

**Conflict of interest:** Carlos-A. Mestres discloses fees from Edwards Clinical Events Committee (CEC) and Cytosorbents Corp. The other authors have nothing to disclose. 

### Reviewer information

Interactive CardioVascular and Thoracic Surgery thanks Lenard Conradi and the other, anonymous reviewer(s) for their contribution to the peer review process of this article.
